# Lipoic Acid Ameliorates Lipopolysaccharide‐Induced Inflammation via Inhibition of Glycolysis in RAW264.7 Macrophages

**DOI:** 10.1002/iid3.70313

**Published:** 2026-01-08

**Authors:** Liduo Yue, Kai Wang, Rongyuan Wang, Linbei Lu, Lihong Fan

**Affiliations:** ^1^ Shanghai Tenth People′s Hospital, Institute of Energy Metabolism and Health, Tongji University School of Medicine Shanghai China; ^2^ Shanghai YangZhi Rehabilitation Hospital （Shanghai Sunshine Rehabilitation Center) Tongji University Shanghai People's Republic of China; ^3^ School of Laboratory Medicine and Biotechnology Southern Medical University Guangzhou China; ^4^ Department of Pulmonology Sixth People′s Hospital affiliated to Shanghai Jiaotong University Shanghai China

**Keywords:** anti‐inflammation, HIF1α, lipoic acid, macrophage

## Abstract

**Background:**

Sustained pulmonary inflammation contributes significantly to lung carcinogenesis. Macrophages play a pivotal role in perpetuating inflammatory responses, undergoing a metabolic shift from oxidative phosphorylation (OXPHOS) to glycolysis upon activation. The interplay between metabolic reprogramming and macrophage polarization remains poorly defined. The objective of this study is to examines the anti‐inflammatory mechanism of lipoic acid (LA), focusing on its ability to modulate immunometabolism in activated macrophages.

**Methods:**

We utilized lipopolysaccharide (LPS)‐stimulated RAW264.7 macrophages and a murine acute lung injury (ALI) model to evaluate the anti‐inflammatory effects of LA. Inflammatory cytokine expression was assessed by qPCR, ELISA, and Western blot. Metabolic profiling was performed using Seahorse XF technology to measure oxygen consumption rate (OCR) and extracellular acidification rate (ECAR), evaluating glycolytic and oxidative metabolic functions.

**Results:**

This study systematically elucidates the molecular mechanism by which LA modulates macrophage inflammatory responses through targeting the HIF1α/glycolysis axis. The main findings are as follows: (1) In LPS‐induced RAW264.7 macrophages, LA treatment significantly inhibited the expression of M1 macrophage markers (iNOS, CD86) and the secretion of proinflammatory cytokines (IL‐1β, IL‐6, etc.). (2) LA effectively reduced the expression of GSDMD, the key executor of pyroptosis, demonstrating its inhibitory effect on macrophage pyroptosis. (3) Metabolic analysis revealed that LA reversed LPS‐induced metabolic reprogramming by decreasing the ECAR and increasing the OCR, thereby suppressing glycolysis. (4) Mechanistic studies showed that siRNA‐mediated knockdown of HIF1α replicated both the anti‐inflammatory and metabolic regulatory effects of LA, confirming HIF1α as the key target in this pathway. (5) In an ALI mouse model, LA treatment significantly reduced HIF1α expression in lung tissues and effectively alleviated inflammatory responses, further validating the proposed mechanism.

**Conclusion:**

LA exerts potent anti‐inflammatory effects by targeting HIF1α‐mediated metabolic reprogramming in macrophages. Our results highlight the therapeutic potential of targeting immunometabolic pathways in inflammatory lung diseases, providing new insights into the mechanism by which LA ameliorates pulmonary inflammation.

## Graphical abstract

Anti‐inflammatory mechanism of Lipoic acid. LA and siRNA‐HIF1α inhibited the expression of HIF1α and inflammatory factors such as IL‐6, IL‐18, TNF‐α, and IL‐1β in LPS‐induced macrophages. The researchers showed in this work that the anti‐inflammation was achieved through HIF1α/glycolysis axis.

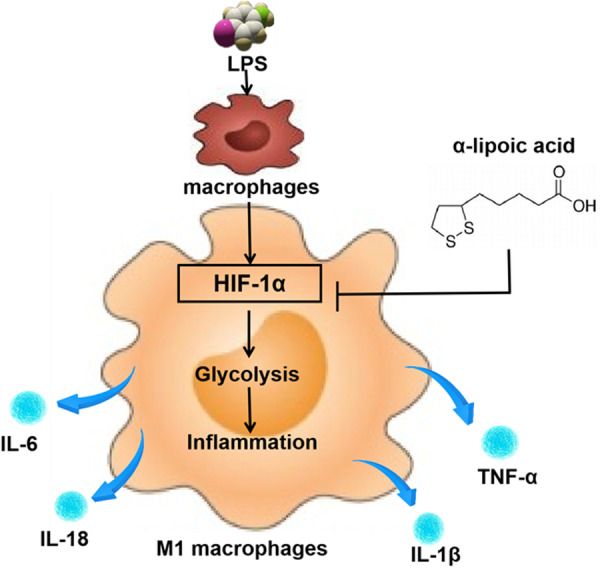

## 1 Introduction

According to recent evidence, inflammation is the seventh most significant hallmark of cancer [1, 2, 3]. Epidemiological studies demonstrate that chronic infections can cause various types of cancer [4], and nonsteroidal anti‐inflammatory drugs have been shown to inhibit a broad spectrum of tumors [5]. Lung cancer, the most prevalent type of primary cancer, has seen a rise in its 5‐year survival rate over the past four decades; however, no significant improvement has been made since the 1980s. Numerous sources indicate that chronic pneumonia is a cause of lung cancer [6]. In chronic pneumonia inflammation, macrophages play a crucial role; continuously activated macrophages produce inflammatory factors and destroy nearby tissues [6].

Macrophages are essential to natural immunity and play a key role in inflammation and host defense [7]. They display functional plasticity in response to microenvironmental cues [8], and they are generally categorized into two broad but distinct subsets: classically activated (M1) or alternatively activated (M2). However, macrophages represent a continuum of highly plastic effector cells, resembling a spectrum of diverse phenotype states [8, 9]. Interferon‐gamma (IFN‐γ) and lipopolysaccharides (LPS) can classically induce M1 polarization of macrophages [10]. M1 macrophages produce proinflammatory factors, including tumor necrosis factor (TNF)‐α, interleukin (IL)‐1β, interleukin (IL)‐6, and interleukin (IL)‐18, and have a potent capacity for antigen presentation capability [11]. This proinflammatory reaction protects the host against pathogens. However, prolonged maintenance of this state can harm the body by creating an inflammatory microenvironment that releases inflammatory factors and reactive oxygen species (ROS), which further damage nearby normal cells [12]. This can result in the malignant transformation, apoptosis, or necrosis of many essential cells, causing tissue and organ lesions that are frequently accompanied by distinct clinical symptoms [13].

Macrophage activation is a two‐phase process. The first phase involves the transcription of inflammatory factors that are not secreted. The second phase is pyroptosis, characterized by the release of large quantities of inflammatory factors, culminating in cell rupture and death. This process amplifies destructive effects [14]. Consequently, inhibiting M1 macrophage polarization and the subsequent release of inflammatory factors in the relatively early stages of chronic inflammation can effectively prevent the initiation of lung tumors.

Hypoxia‐inducible factors (HIFs) are a family of transcription factors essential for cell adaptation to low oxygen conditions. The alpha subtype, HIF1α, is known to regulate glucose metabolism [4]. Specifically, HIF1α can promote the expression of glycolytic enzymes [15, 16]. It is also recognized as a key regulator in inflammatory cells, where it enhances the expression of inflammatory genes [17, 18, 19]. Given the crucial role of HIF1α in both glycolysis and the inflammatory response, it is important to investigate how it regulates macrophage function during inflammation. M1 macrophages predominantly rely on glycolysis as their energy metabolic pathway [20, 21]. It has been hypothesized that a metabolic switch from oxidative phosphorylation (OXPHOS) to aerobic glycolysis is a defining feature of M1 polarization [22, 23, 24]. Alpha‐lipoic acid (LA) is an antioxidant present in all prokaryotic and eukaryotic organisms. As an essential cofactor of the mitochondrial respiratory chain, such as pyruvate dehydrogenase (PDH), LA plays a crucial role in human energy metabolism [18]. It has also been reported that LA modulates inflammatory response, exerts beneficial effects in various cancers, and contributes to the effective treatment of chronic conditions [25, 26, 27]. For example, LA can inhibit LPS‐induced inflammatory responses via the PI3K/Akt pathway [28]. By suppressing ERK, p38, and NF‐κB signaling, LA also prevents the release of TNF‐α from histone‐stimulated macrophages [29, 30, 31, 32, 33]. Additionally, LA inhibits the production of nitric oxide (NO) and TNF‐α in LPS‐induced rat Kupffer cells and RAW 264.7 mouse macrophages [34, 35, 36, 37, 38]. However, the precise mechanism by which LA regulates LPS‐stimulated macrophages remains unclear.

In this study, we demonstrate that LA exerts potent anti‐inflammatory effects by targeting HIF1α‐mediated metabolic reprogramming in macrophages, as evidenced by experiments conducted in LPS‐stimulated RAW264.7 cells and a murine model of acute lung injury (ALI). These findings provide a mechanistic basis for the development of LA‐based interventions aimed at mitigating inflammation‐driven tumorigenesis, offering potential novel strategies for anti‐inflammatory prevention and treatment.

## 2 Materials and Methods

### 2.1 Reagents

LPS (Cat. No. L2880) was purchased from Sigma‐Aldrich (USA). LA (Cat. No. Hy‐N0492) and 2‐deoxygenated glucose (2‐DG, Cat. No. 13966) were purchased from MedChemExpress (USA); HIF1α (Cat. No. 3716), p‐AMPK (Cat. No. 2535), AMPK (Cat. No. 2532), Cleaved Gasdermin D (Cat. No. 10137), and β‐actin (Cat. No. 5125) antibodies were purchased from the Cell Signaling Technology (USA). Brilliant Violet 421 anti‐mouse CD86 (Cat. No. 305426). The antibody was from Biolegend (USA). MitoSox probe (Cat. No. M36008) and Dulbecco′s Modified Eagle Medium (DMEM, Cat. No. C11995500) were from Thermo Fisher Scientific (USA). ELISA kit for interleukin (IL)‐1β (Cat. No. ab100704), interleukin (IL)‐6 (Cat. No. ab222503), and interleukin (IL)‐18 (Cat. No. ab216165), and lactate (Cat. No. ab65330) were purchased from Abcam Plc (UK).

### 2.2 Cell Culturing and LPS Treatments

RAW246.7 cell line (Cat. No. ZQ0098) purchased from Shanghai Zhongqiao. com, Shanghai, China) were cultured in DMEM medium containing 10% FBS (Cat. No. C0232, GIBCO, USA) and 1% penicillin‐streptomycin solution (Cat. No. SV30010, Hyclone, USA) in a 37°C, 5% CO_2_ incubator. LPS (Sigma, Cat. No.4395) were diluted in DMEM medium. After cells were seeded into six wells for 24 h, the cells were exposed to diluted LPS to establish the inflammatory cell model.

### 2.3 Quantitative Real‐Time Polymerase Chain Reaction (qRT‐PCR)

The Invitrogen Life Technologies TRIzol Reagent was used to extract total RNA, which was then quantified and analyzed for quality. Complementary DNA (cDNA) was synthesized using oligo(dT) as a primer and AMV as reverse transcriptase, subsequent to total RNA extraction. RT‐mediated real‐time PCR was carried out with the SYBR green‐dyed ABI system. PCR primer was designed and synthesized by Qingke Biotechnology Co. Ltd. (Beijing, China). Table 1 shows the primer sequences of RNA. GAPDH was used as the internal reference. The relative abundance of mRNAs was calculated by the comparative Ct method [39, 40].

**Table 1:** Primer sequences of RNA.

Gene	Primer sequence (5' to 3')	Annealing (°C)	Product length (bp)	PrimerBank ID
IL‐1β	TGCCACCTTTTGACAGTGATG	60.2	136	NM_008361
	TGATGTGCTGCTGCGAGATT			
IL‐18	ACTTTGGCCGACTTCACTGT	61.8	202	NM_008360
	GTCTGGTCTGGGGTTCACTG			
IL‐6	GGCGGATCGGATGTTGTGAT	62,5	199	AJ345034
	GGACCCCAGACAATCGGTTG			
GAPDH	GTGCAGTGCCAGCCTCGTCC	61.9	280	NM_013527
	AACGCAGCTCAGTAACAGTCC			

### 2.4 Cell Viability Assay

The CCK‐8 kit was used to detect cell viability. (Cat. No. 40203ES60, Yeasen, Shanghai, China). RAW264.7 cells were seeded at 5 × 10^3^ cells/well into 96‐well plates. Cells were cultured for 24 h and treated with different concentrations of LA. Each well was then incubated with 10 μL CCK8 reagents at 37°C in an incubator for 1.5 h, and the optical density (OD) was then measured. Cell viability (%) = (experiment group OD‐blank medium OD)/(DMSO control group OD—medium OD) [4].

### 2.5 Western Blotting Analysis

Cells in six‐well plates (about 5 × 10^5^ cells/well) were washed with PBS buffer, harvested in RIPA buffer, and incubated for 30 min on ice. The protein‐containing supernatant was extracted by centrifuging lysates at 12,000*g*, 10 min, and 4°C. Add 20 μg protein sample per well, 80 V constant‐voltage electrophoresis for 30 min, and then convert to 120 V constant voltage electrophoresis for protein separation on a 10% SDS‐polyacrylamide gel. After 2 h of 200 mA constant flow film, the immunoblot was blocked for 1 h with 5% BSA, washed three times with PBS, and incubated overnight at 4°C with HIF1α, p‐AMPK, and Cleaved Gasdermin D (the pyroptosis marker) antibodies. Three times 15 min washes with TBST were followed by a 1‐h incubation with a 1:5000 dilution of HRP‐conjugated secondary antibody. The blots were washed thrice for 15 min with TBST, then enhanced chemiluminescence was used to develop them. The protein band intensity from Western blotting was calculated by Image J software [41].

### 2.6 Cell Transfection and siRNA Analysis

RAW264.7 were seeded in serum‐free medium 12 h after LPS stimulation. Three siRNAs (siRNA‐1, siRNA‐2, and siRNA‐3) targeting HIF1α were designed and synthesized, along with a nonspecific siRNA (NC) and a GAPDH siRNA (siRNA‐NC), and then were transfected into distinct groups of RAW264.7 cells. siRNA‐2 was determined to be the most efficient HIF1α siRNA. The sequences of the three HIF1α siRNAs were as below:

siRNA‐1: 5′‐ GCUGACCAGUUACGUUACGAUUGUTT‐3′;

siRNA‐2: 5′‐ CUGAUAACGUGAACAAAUATT‐3′; and

siRNA‐3: 5′‐ GACACAGCCUCGAUAUGAATT‐3′;

Six hours after transfection, the transfected cells were fed with fresh medium and incubated for 24 h. Finally, gene silencing was confirmed by measuring the expression of HIF1α protein using western blotting, and the most efficient siRNA was figured out for the remaining experiments.

### 2.7 HIF1α Overexpression

The HIF1α plasmids were acquired from Hanheng Biotechnology. The cells were mixed with the transfection mixture (Lipofectamine2000 with a vehicle vector or HIF1α plasmid) either with or without LA exposure. After 6 h of incubation, the cells were rinsed and incubated for an additional 24 h before further examination [42].

### 2.8 Flow Cytometry Analysis for Mitochondrial ROS

For MitoSOX staining, cells were cultured at 37°C for 10 min in a buffer containing 10% serum and 2.5 M MitoSOX Red. The cells were then trypsinized, rinsed once with cold PBS, and resuspended in PBS containing 0.1% serum. MitoSOX samples were immediately analyzed with the BD FACSFertessa Cytometry System (BD Biosciences). Before measuring fluorescence, cells were gated by size using FSC and SSC, then by FSC height x FSC area to exclude doublets. Globally, the intensity threshold was established at 10% of the control sample containing positive cells (without LA). The cell with a signal beyond the threshold was regarded as the MitoSOX‐positive cells, using the positive percentage to make the bar graphs. MitoSOX quantification methods were performed according to the methods described [43].

### 2.9 Flow Cytometry Analysis for M1 Macrophages Phenotypes by Testing Surface Molecules CD86

Trypsin/EDTA solution was used to collect differentiated cells at 37°C for 5 min, followed by three washes with PBS. Then, the cells were incubated with anti‐mouse CD86 antibody at 4°C for 30 min. Next, a flow cytometry buffer solution was used to wash the cells. After 30 min of fixation with PBS, flow cytometry was used to detect the expression of CD86. The data were analyzed utilizing the FlowJo software [44].

### 2.10 Measurement of Oxygen Consumption Rate (OCR) and Extracellular Acidification Rate (ECAR)

Usually, OCR reflects mitochondrial OXPHOS ability, and ECAR reflects cell glycolysis level. According to the manufacturer, OCR and ECAR were measured using a seahorse Bioscience XF24 extracellular flux analyzer (Agilent Technologies, Palo Alto, CA, USA). Forty‐eight hours before the assay, Raw264.7 cells were seeded at a density of 20,000 cells per well in XF microplates (Seahorse Bioscience, Billerica, MA, USA) containing DMEM medium containing 10% FBS. A 24‐h treatment with 200, 400 μM LA was carried out.

Before the Mito Stress Test, the medium was replaced with XF base medium (Seahorse Bioscience, Billerica, MA, USA) containing 1 mM Pyruvate, 2 mM Glutamine, and 10 mM Glucose and incubated at 37°C without CO_2_ for 1 h. Using a Seahorse XF analyzer, OCR was measured during the test. According to the manufacturer's instructions, a Mito Stress Test Kit containing 1 M Oligomycin, 1 M Carbonyl cyanide‐4‐(trifluoromethyl) phenylhydrazone (FCCP), and 0.5 M Rotenone/0.5 M Antimycin A was used.

Prior to Glycolytic Rate Assay, the medium was replaced with XF base medium without phenol red (Seahorse Bioscience, Billerica, MA, USA) containing 2 mM glutamine, 10 mM glucose, 1 mM pyruvate, and 5 mM HEPES and incubated at 37°C without CO_2_ for 1 h. ECAR was measured during the test using a Seahorse XF analyzer. According to the manufacturer′s instructions, a Glycolytic Rate Assay Kit comprised of 0.5 M Rotenone, 0.5 M Antimycin A, and 50 mM 2‐Desoxyglucose was utilized. After the assay, ECAR and OCR were calculated and normalized to cell numbers using Wave 2.6 (Seahorse Bioscience, Billerica, MA, USA) [40].

### 2.11 Animal Experimentation and Grouping

Six‐week‐old male C57BL/6 black mice (Shanghai Laboratory Animal Center, Shanghai, China) were housed in a conventional animal laboratory, fed a standard diet (the diet was according to Chinese General quality STANDARD for formula animal feeds—GB14924.1) and had free access to water. The animal experiment was carried out with the approval of the Experimental Animal Ethics Committee of Shanghai 10th People′s Hospital, Tongji University School of Medicine (approval number: SHDSYY2020–3289, Shanghai, China). As previously depicted, the LPS‐induced in vivo ALI model was established. After the accommodation to the SPF‐barrier facility for 7 days, 25 mice were equally and randomly divided into five groups: control groups (PBS), LPS (50 μg/mL × 100 μL), LPS (150 μg/mL × 100 μL), and LA + LPS (150 μg/mL × 100 μL LPS + 90 mg/kg LA and positive control (150 μg/mL × 100 μL LPS + 10 mg/kg Dexamethasone) groups. LA and Dexamethasone were administered by intra‐abdominally 9 h after LPS inhalation. All animals were narcotized with an injection of Pentobarbital sodium solution and killed 24 h later by dislocation. The lung was removed, embedded in paraffin, fixed in 4% paraformaldehyde for 24 h, and sectioned for Histopathologic assay, Western blotting, and RT‐qPCR assay [39, 45, 46].

### 2.12 Histopathologic Assay

Lungs were sectioned in at least three planes and stained with hematoxylin and eosin (HE) at a thickness of 2 µM. The lung tissue stained by HE was photographed using the fluorescence microscope with the digital vidicon (DM 4000B, Leica, Germany).

IHC‐stained lung tissues were choped into 4‐μm thick profiles, which were dyed with anti‐HIF1a antibody, incubated with the appropriate secondary antibody, and photographed under the microscope furnished with the digital vidicon. The histology score (H score) was numbered on the basis of the dying intensity and the percentum of dyed cells. Comparable with stromal cells, the intensity score of the stained cells was defined as such: 0, no appreciable dying; 1, weak dying; 2, intermediate dying; and 3, strong dying. The portion of positively dyed cells was recorded as 0%–100%, comparable with stromal cells. By the multiplication of the intensity scores and the portion scores, the H score of the stained cells was calculated and produced the overall scope of 0–300 for quantitative HIF1a based on the staining intensity and the proportion of stained cells. Two independent researchers without knowledge of the grouping examined and scored tissue sections separately [47].

### 2.13 ELISA for Cytokine Detection in Cell Culture Supernatant and Plasma

Concentrations of IL‐1β, IL‐6, and IL‐18 in cell culture supernatant and plasma samples were determined using commercial ELISA kits (Abcam, catalog no. ab100704, ab222503, and ab216165) following the manufacturer′s instructions. In brief, 100 μL of standards and the collected cell culture supernatant were added in duplicate to the pre‐coated 96‐well microplate and incubated for 2.5 h at room temperature. After incubation, the liquid was aspirated, and wells were washed four times with 300 μL of 1× wash buffer. Following the last wash, residual liquid was thoroughly removed by aspiration. Then, 100 μL of 1× biotinylated anti‐IL‐1β (or IL6, IL18) detection antibody was added to each well, and the plate was incubated for 1 h at room temperature under gentle shaking. Subsequent steps including streptavidin‐HRP incubation, substrate reaction, and stop solution addition, were carried out exactly as described in the kit protocol. Absorbance was measured at 450 nm using a microplate reader. A standard curve was generated from the reference standards, and sample concentrations were interpolated using the curve.

### 2.14 Statistical Analysis

GraphPad Prism 8.0 (GraphPad software) was used for statistical analysis. Three independent experiments were performed to generate raw data. A one‐way ANOVA test was used for comparison of more than two groups, with Tukey or Sidak′s test for multiple comparisons. A two‐tailed Student′s *t*‐test was used when there were only two groups for analysis. A *p*‐value < 0.05 was considered significant (*), ** indicates *p* < 0.01; *** indicates *p* < 0.001.

## 3 Results

### 3.1 LA Decreases Proinflammatory Cytokine Production and HIF1α Expression in LPS‐Induced RAW264.7 Macrophages and Increases Pampk Expression

**Figure 1:**
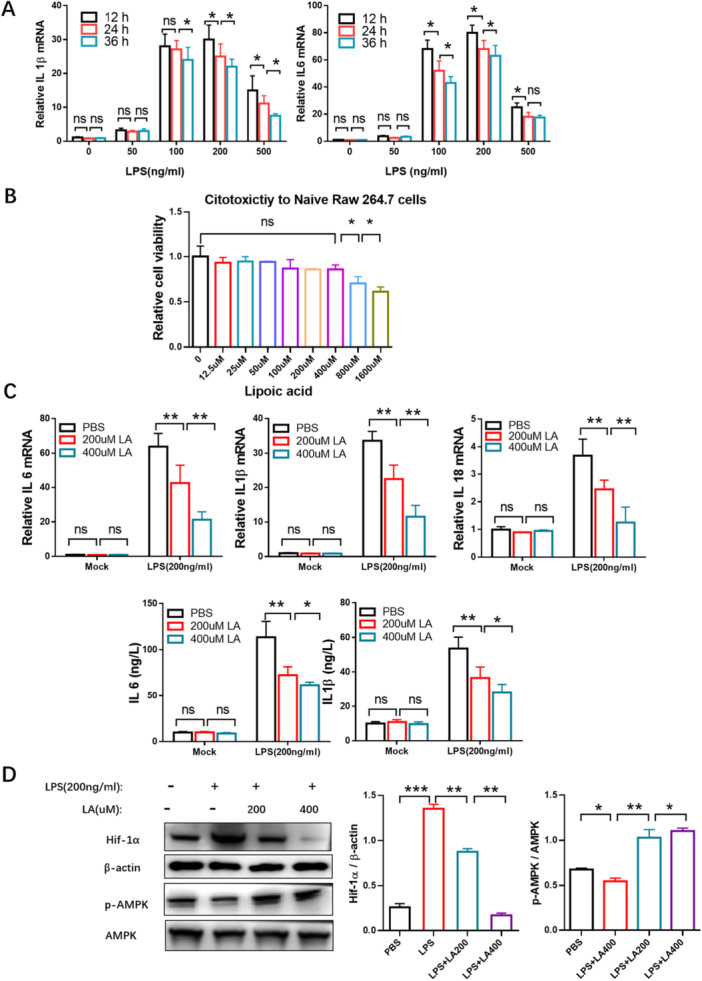
Effect of LA on inflammation of LPS‐induced RAW264.7 cells and the expression of HIF1α and p‐AMPK. (A) The inflammatory factors IL‐1β and IL‐6 were assessed using RT‐qPCR to figure out LPS concentration for establishing the inflammatory cell model. RAW264.7 cells were treated with increasing concentrations of LPS (0, 50, 100, 200, and 500 ng/mL) for different time incubation (12, 24, and 36 h). (B) Cytotoxic effect of LA on RAW264.7 cells was evaluated via CCK‐8 assays. (C) The anti‐inflammatory effect of LA on IL‐1β, IL‐6, and IL‐18 expression of 200 ng/mL LPS‐induced RAW264.7 cells was investigated using RT‐qPCR or in the supernatants by ELISA. (D) The effect of LA on the expression of mitochondrial metabolic involved HIF1α and p‐AMPK was determined by western blotting. M0, untreated RAW264.7; M1, RAW264.7, treated with LPS. Results were shown as the mean ± s.d. The symbol * indicates *p* < 0.05, ** indicates *p* < 0.01, *** indicates *p* < 0.001.

To establish an inflammatory model using RAW264.7 cells, the macrophages were exposed to increasing concentrations of LPS (0, 50, 100, 200, and 500 ng/mL LPS) for 12, 24, and 36 h. The expression of proinflammatory cytokine genes was assessed using real‐time quantitative PCR (RT‐qPCR). Treatment with 200 ng/mL LPS resulted in the highest production of IL‐1β and IL‐6. Inflammatory factor expression was more pronounced at 12 h compared with 24 or 36 h (Figure 1A). Therefore, incubation with 200 ng/mL LPS for 12 h was selected as the optimal condition for establishing the inflammatory macrophage model.

To evaluate the cytotoxicity of LA on RAW264.7 cells, cell viablility was measured at various concentrations of LA using a CCK‐8 assays across a range of LA concentrations. LA concentrations up to 400 µM showed no cytotoxic effects (Figure 1B). Based on these results, 200 µM, 400 µM LA were used to assess their impact on the expression of proinflammatory cytokines IL‐1β, IL‐6, and IL‐18 via RT‐qPCR, as well as IL‐6 and IL1β levels in the supernatants via ELISA. The results demonstrated that LPS activation increased the levels of IL‐1, IL‐6, and IL‐18, which were subsequently reduced upon treatment with LA at both concentrations (Figure 1C).

Western blot analysis showed that LPS induction upregulated HIF1α expression in RAW264.7 cells, which was suppressed by LA in a dose‐dependent manner. In contrast, phosphorylated‐AMPK (pAMPK)—known to promote oxidative phosphorylation (OXPHOS) [48]—was downregulated following LPS stimulation and upregulated after LA treatment. Total AMPK levels remained unchanged (Figure 1D). Although both HIF1α and pAMPK were modulated by LA in LPS‐induced RAW264.7 cells, the causal relationship between HIF1α and pAMPK remains unclear.

### 3.2 LPS Induced‐RAW264.7 Cells Exhibit M1 Macrophage Phenotypes and Pyroptosis Character, and LA Can Inhibit the M1 Phenotypes and Pyroptosis

**Figure 2:**
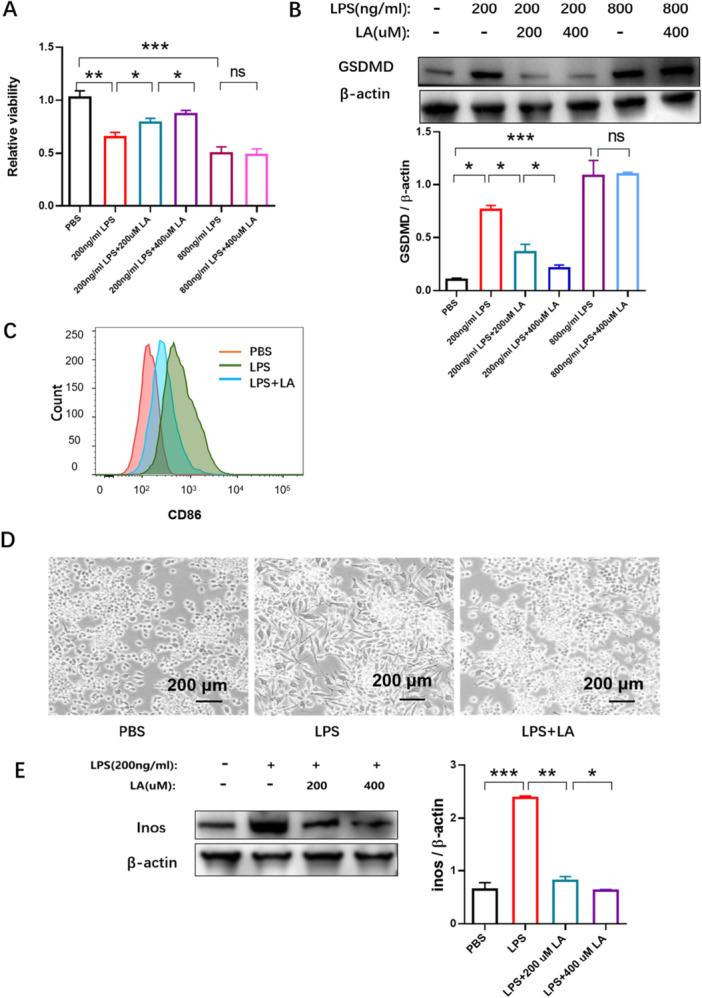
Inhibitory effect of LA on M1 macrophage phenotypes. (A) Cell viability of LPS‐induced RAW264.7 macrophages after treatment with LA was investigated using CCK8 assays. (B) The effect of LA on the expression of pyroptosis marker protein, GSDMD, was detected by western blotting. (C) LA lowered the expression of the M1 macrophage surface marker CD86, determined by flow cytometry. (D) Cell morphology of RAW264.7 cells exposed to 200 ng/mL LPS and ±400 µM LA were captured under the bright field of the microscope (Scale bar = 200 μm). (E) LA reduced the expression of the M1 marker, iNOS, determined by western blotting. Results were shown as the mean ± s.d. The symbol * indicates *p* < 0.05, *** indicates *p* < 0.001.

It has been previously reported that LPS reduces the viability of RAW264.7 cells and exerts a proinflammatory effect [4, 49]. After treatment with LA, the viability of LPS‐induced‐RAW264.7 cells increased in a dose‐dependent manner (Figure 2A). However, when RAW264.7 cells were treated with 800 ng/mL LPS, cell viability decreased dramatically and could not be restored by LA (Figure 2A). The pyroptosis marker, cleaved Gasdermin D (GSDMD), was detected by western blotting to evaluate the level of pyroptosis. Induction of GSDMD expression was greater at 800 ng/mL LPS than at 200 ng/mL (Figure 2B). LA ameliorated pyroptosis induced by 200 ng/mL LPS but not 800 ng/mL LPS. Flow cytometry analysis showed that 200 ng/mL LPS also induced expression of the CD86 antigen marker (an M1 phenotype) on the surface of RAW264.7 macrophages, and LA was able to reverse this M1 polarization (Figure 2C). Western blot analysis further revealed increased expression of the inducible nitric oxide synthase (iNOS, an M1 macrophage marker) in LPS‐induced RAW264.7 cells (Figure 2D). LA inhibited the expression of both CD86 and iNOS, indicating that it promoted a shift from the LPS‐induced M1 phenotype back to the untreated M0 state (Figure 2C,D). After stimulation with 200 ng/mL LPS, light microscopy showed an increase in pseudopodia formation, as well as elongation and flattening of RAW264.7 cells. Following incubation with LA, the number of pseudopodia decreased, and the cells gradually returned to a round morphology, suggesting that LA can alter the morphology of LPS‐induced macrophages (Figure 2E).

### 3.3 LA Inhibits Glycolytic Metabolism in LPS‐Induced RAW264.7 Macrophages

**Figure 3:**
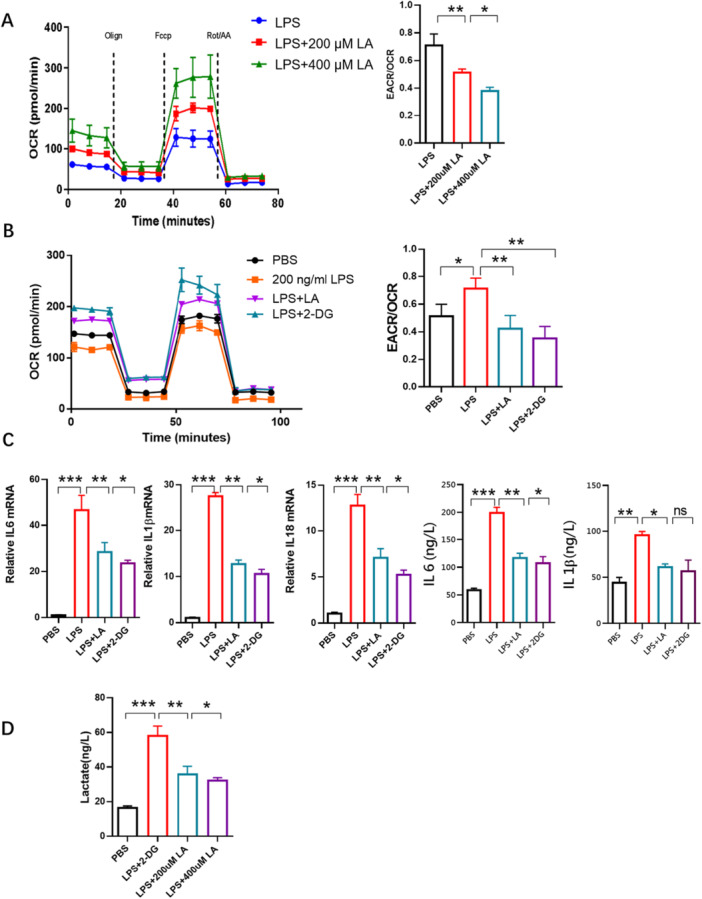
Effect of LA on energy metabolism phenotype of LPS‐induced RAW264.7 cells. Extracellular acidification rate (ECAR) and oxygen consumption rate (OCR) were investigated using the XF Seahorse apparatus. (A) LA dose‐dependently reduced the level of glycolysis, reflected by increased OCR and decreased ratio of ECAR/OCR. (B) 2‐DG, a glycolytic enzyme inhibitor, inhibited glycolysis, like LA. (C) 2‐DG and LA reduced the production of IL‐1β, IL‐6, and IL‐18 monitored by RT‐qPR or IL‐1β, IL‐6 in the supernatants by ELISA. (D) Extracellular lactate was determined by ELISA. Results were shown as the mean ± s.d. The symbol * indicates *p* < 0.05, ** indicates *p* < 0.01, *** indicates *p* < 0.001.

Activation macrophages (M1macrophages) are characterized by enhanced glycolysis and a disrupted TCA cycle, leading to metabolite accumulation and chronic inflammation [50]. In this study, we used the XF Seahorse analyzer to examine the effect of LA on the metabolic phenotype of LPS‐induced RAW264.7 macrophages (M1). LA treatment increased the OCR and decreased the ECAR in a dose‐dependent manner, indicating that LA suppresses glycolysis in M1 macrophages (Figure 3A). The reduced ECAR/OCR ratio suggested a metabolic shift from glycolysis toward oxidative phosphorylation (OXPHOS). The hexokinase inhibitor 2‐deoxyglucose (2‐DG), known to inhibit glycolysis, was used as a reference. Treatment of M1 cells with 2‐DG similarly increased OCR and decreased the ECAR/OCR ratio (Figure 3B). RT‐qPCR and ELISA results showed that 2‐DG, like LA, suppressed the expression of inflammatory factors (Figure 3C), suggesting that inhibition of glycolysis is positively correlated with anti‐inflammatory effect (Figure 3B,C). Lactate levels in culture supernatant of LPS‐induced RAW264 cells were measured using ELISA. The results showed that LPS increased lactate production (a glycolysis product) and subsequent LA treatment reversed this effect (Figure 3D). Together, these results indicate that that LA acts as a glycolysis inhibitor and attenuates the inflammatory response by suppressing glycolysis.

### 3.4 LA Inhibits Glycolysis and the Inflammatory Response of LPS‐Induced Macrophages via Inhibiting HIF1α

We have previously confirmed that LA can reduce the inflammatory response and suppress glycolysis of M1 macrophages; LA decreased HIF1α and increased pAMPK expression in LPS‐induced RAW 264.7 macrophages. Since HIF1α and phosphorylated‐AMPK are involved in the transition from glycolysis to OXPHOS [51, 52, 53, 54]. We were curious which factor plays a dominant role: HIF1α or pAMPK? Does LA primarily inhibit glycolysis and inflammation in M1 cells by suppressing HIF1α or by activating pAMPK?

**Figure 4:**
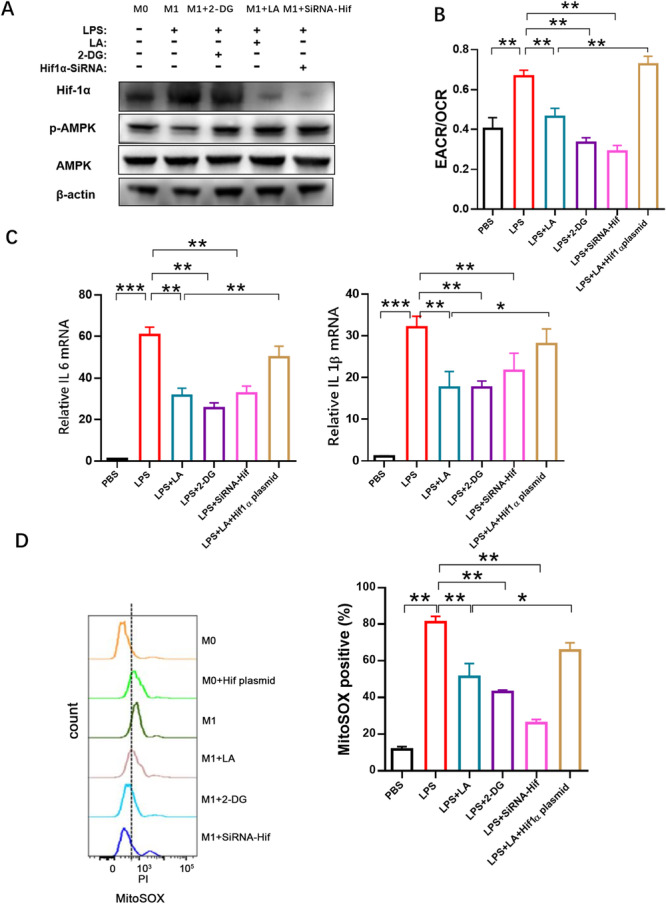
LA inhibited the inflammatory response of LPS‐stimulated RAW264.7 macrophages by inhibiting HIF1α, and glycolysis. (A) The effect of siRNA‐HIF1α, 2‐DG, and LA on the expression of HIF1α, β‐actin, p‐AMPK, and whole AMPK were determined by Western blotting. (B) The level of glycolysis decreased with the inhibition of HIF1a pathway by monitoring ECAR/OCR ratio on the seahorse apparatus. (C) Overexpression of HIF1α increased levels of inflammatory factors, and inhibition of HIF1α reduced the expression of inflammatory factors. (D) Mitochondrial ROS was monitored with MitoSOX probe by flow cytometry. Results were shown as the mean ± s.d. The symbol * indicates *p* < 0.05, ** indicates *p* < 0.01, *** indicates *p* < 0.001.

Western blot was used to measure the expression of HIF1α and pAMPK after treatment wtih LPS, LA, 2‐DG, and siRNA‐HIF1α (Figure 4A). The results showed that the glycolysis inhibitor 2‐DG increased pAMPK expression but had no effect on HIF1α expression, suggesting that HIF1α acts upstream of glycolysis, while pAMPK is downstream. The promotion of pAMPK‐mediated OXPHOS following glycolysis inhibition was supported by a low ECAR/OCR ratio upon 2DG treatment (Figure 4B). Small interfering RNA targeting HIF1α (siRNA‐HIF1α) inhibited HIF1α and promoted pAMPK expression, indicating that HIF1α suppression leads to glycolysis inhibition and OXPHOS promotion. The ECAR/OCR ratio in LPS‐stimulated RAW264.7 macrophages was increased by HIF1α overexpression and decreased by 2‐DG, LA, and siRNA‐HIF1α (Figure 4B), further supporting that LA, like other HIF1a inhibitors, suppresses glycolysis by inhibiting HIF1a. Moreover, the inhibitory effect of LA on glycolysis could be reversed by HIF1α overexpression, indicating that LA inhibits glycolysis and inflammation in LPS‐stimulated RAW264.7 cells primarily through HIF1α inhibition (Figure 4B).

In addition, the release of inflammatory factors induced by HIF1α overexpression was suppressed by LA, siRNA‐HIF1α, or 2‐DG, consistent with the inhibition of glycolysis and subsequent reduction in inflammation levels (Figure 4C).

ROS produced by mitochondrial OXPHOS often reflect an inflammatory response. In this study, ROS levels were monitored using a MitoSOX probe in the flow cytometry assays. ROS increased in response to LPS and were inhibited by LA, 2‐DG, and siRNA‐HIFα (Figure 4D).

In conclusion, by inhibiting the HIF1α‐induced glycolysis, LA downregulates the inflammatory response of LPS‐stimulated RAW264.7 macrophages.

### 3.5 LA Inhibits Pulmonary Inflammation and Reduces the Level HIF1α In Vivo

**Figure 5:**
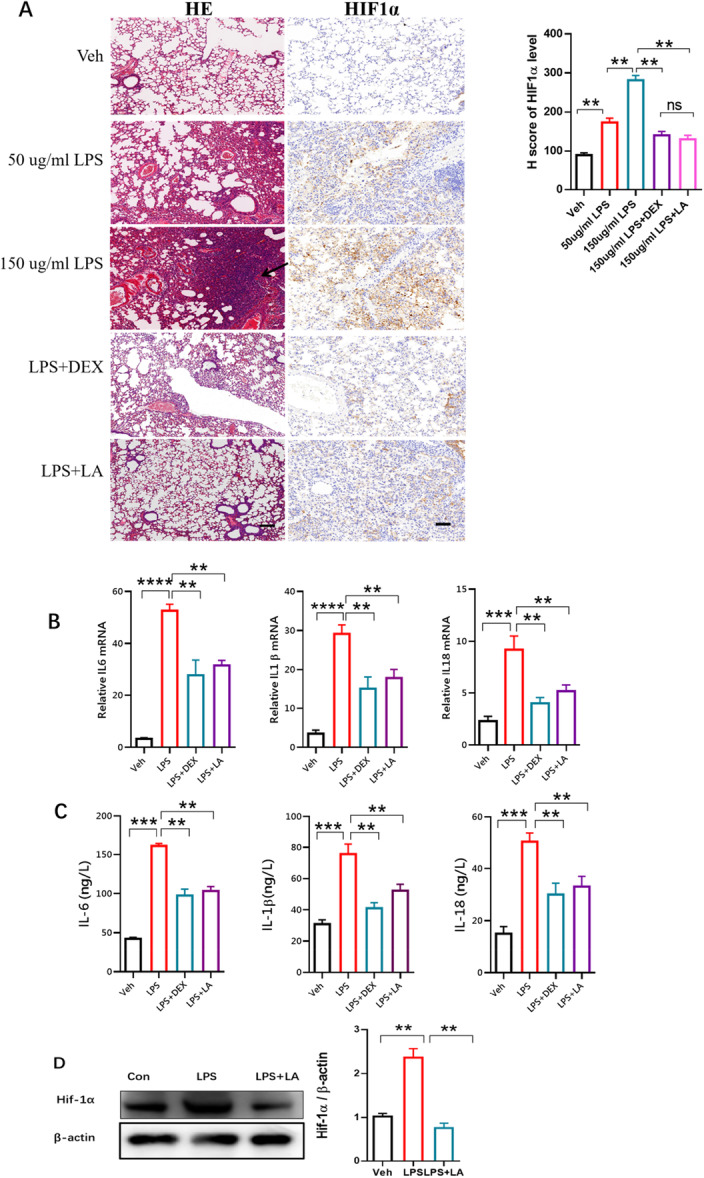
Anti‐inflammatory effect of LA on LPS‐stimulated acute inflammation in mice. (A) After inhaling 100 μL LPS(50 μg/mL, 150 μg/mL), the lung exhibited inflammation, as shown by HE staining, and this inflammation was ameliorated after Dexamethasone (a NSAID as positive control) or LA treatment (Scale bar = 100 μm). (B) After LA treatment, inflammatory cytokines in pulmonary tissue were analyzed by RT‐qPCR. LA can inhibit pro‐inflammatory cytokines induced by LPS. (C) The serum levels of inflammatory factors were decreased after LA treatment evaluated by ELISA assays. (D) Expression of HIF1α in the lung tissue of mice was decreased after LA treatment. (LPS in B–D: 150 µg/mL × 60 µL; LA:91 mg/kg); Con, without LPS and LA treatment. Results were shown as the mean ± s.d. The symbol * indicates *p* < 0.05, ** indicates *p* < 0.01, *** indicates *p* < 0.001, **** indicates *p* < 0.0001.

To investigate the anti‐inflammatory effect of LA in vivo, we used C57BL/6 mice with ALI induced by LPS. HE staining demonstrated that LPS triggered an acute inflammatory response in the lung tissue compared with the untreatment group, with inflammatory features indicated by black arrows. The inflammatory symptoms were significantly alleviated by treatment with either dexamethasone or LA (Figure 5A). LA exhibited an anti‐inflammatory effect comparable to that of dexamethasone. HIF1a expression was also examined by IHC assay, and the results were quantified in H‐score. The data revealed that HIF1α was upregulated by LPS and downregulated after LA treatment. Furthermore, RT‐qPCR analysis indicated that LPS stimulation increased the expression of inflammatory factors in lung tissue, while LA treatment reduced their expression (Figure 5B). Serum was extracted by centrifuging blood collected from mouse eyeballs. ELISA results showed that LA treatment resulted in low levels of inflammatory factors in the serum of mice (Figure 5C). Western blot analysis of lung tissues reveals that the expression of HIF1α was increased following LPS stimulation and decreased after LA treatment (Figure 5D). These results indicate that LA suppresses HIF1α expression and exerted an anti‐inflammatory effect in the ALI animal model, which supports the findings from the cell‐based experiments in previous sections.

## 4 Discussion

Macrophages are the primary inflammatory cells in the body. They serve not only as a crucial defense mechanism against infections but are also deeply implicated in the pathogenesis of numerous diseases. For example, in acute infections and sepsis caused by Gram‐negative bacteria, macrophages become overactivated and release excessive amounts of inflammatory factors, leading to tissue and organ dysfunction—a significant cause of sudden death. Macrophage infiltration and M1 polarization are also observed in various chronic conditions, including obesity, coronary heart disease, arthritis, type 2 diabetes, alcoholic liver damage, atherosclerosis, Alzheimer′s disease, and tumors. Therefore, identifying novel anti‐inflammatory agents that specifically target inflammatory macrophages is of vital importance.

LPS‐activated macrophages, also known as M1 macrophages, produce proinflammatory factors, such as IL‐1β, IL‐6, and IL‐18. These cells also highly express CD86 and iNOS. iNOS generates large amounts of NO, which contributes to the activation of inflammatory signaling pathways. LA is a natural compound known to have fewer side effects than conventional nonsteroidal anti‐inflammatory drugs. In this study, we demonstrated that LA reduced the expression of iNOS, CD86, and proinflammatory cytokines in M1 macrophages. Furthermore, it inhibited ROS production in M1 macrophages and attenuated the inflammatory response in an ALI model. We also found that LPS stimulation decreased macrophage viability, whereas LA treatment mitigated LPS‐induced cytotoxicity and restored cell viability. These results suggest that LA has a protective effect on macrophages.

As indicated by increased levels of aerobic glycolytic enzymes and decreased in oxidative phosphorylation (OXPHOS), M1 macrophages undergo metabolic reprogramming. Inhibiting this metabolic reprogramming has been shown to effectively suppress the inflammatory response [55, 56]. In this study, we confirmed that LPS‐stimulated macrophages exhibit alterations in energy metabolism, which is consistent with previous findings. Following LPS stimulation, the OCR of macrophages decreased, while the ECAR increased, indicating a shift toward glycolysis as the preferred energy metabolism mode characteristic of the M1 phenotype. Glycolysis inhibitors, such as 2‐DG, have been shown to prevent inflammatory factors secretion in M1 cells [57]. We found that LA, similar to 2‐DG, reversed this metabolic pattern in M1 macrophages by increasing OCR and consequently inhibiting glycolysis. In conclusion, LA reduces the inflammatory response both in vitro and in vivo by suppressing glycolytic activity in M1 macrophages.

Pyroptosis, also referred to as inflammatory cell necrosis, is a type of programmed cell death characterized by cellular swelling until membrane rupture occurs, leading to the release of cellular contents that provoke a strong inflammatory response. This process often takes place during cytokine storms [37]. Cleaved gasdermin D (GSDMD), a key marker of pyroptosis, forms pores in the cell membrane that facilitate the release of cytokines [58]. In this study, we observed that LPS stimulation promoted GSDMD production.

Following treatment with a high concentration of LPS (800 ng/mL), the expression of GSDMD was significantly upregulated. While LA was able to inhibit GSDMD expression induced by low doses of LPS, it showed no inhibitory effect under high‐dose LPS conditions. This may be attributed to the fact that LA′s ability to modulate metabolic patterns is insufficient to counteract cell collapse resulting from pyroptosis. Therefore, we hypothesize that LA exerts a more potent anti‐inflammatory effect during the early stages of inflammation, before macrophages undergo pyroptosis. However, this conclusion requires further validation through in vivo studies.

**Figure 6:**
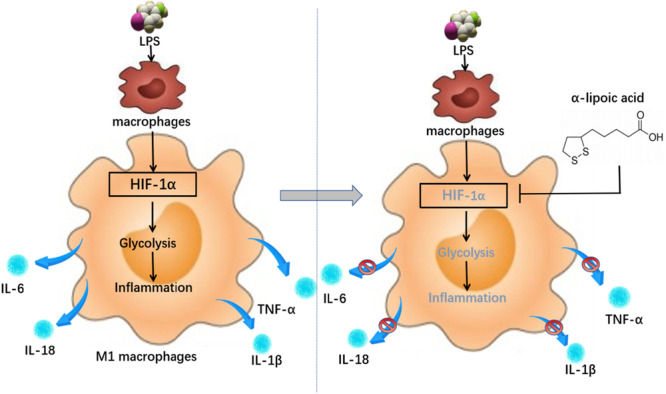
Anti‐inflammatory mechanism of Lipoic acid. LA and siRNA‐HIF1α inhibited the expression of HIF1α and inflammatory factors such as IL‐6, IL‐18, TNF‐α and IL‐1β in LPS‐induced macrophages. The anti‐inflammation was executed through HIF1α/glycolysis axis.

Furthermore, we confirmed the previously reported upregulation of HIF1α expression in macrophages induced by LPS, using both inflammatory RAW264.7 cells and an in vivo ALI model [4]. RT‐qPCR results showed that both LA and HIF1α siRNA inhibited the expression of HIF1α and inflammatory factors in LPS‐induced macrophages. The Seahorse assay demonstrated that both HIF1α siRNA and LA reduced glycolysis in macrophages. These findings suggest that the anti‐inflammatory mechanism of LA operates through the HIF1α/glycolysis axis, as summarized in Figure 6. Therefore, LA may be utilized to alleviate early inflammation, prevent its propagation, and inhibit the progression of inflammatory conditions into tumors and other diseases. However, it remains unclear whether LA can directly bind to HIF1α protein. Clarification of this interaction will be the focus of future work using surface plasmon resonance (SPR), molecular docking, and pull‐down assays.

LA exhibits a wide range of therapeutic effects. It has been reported to function both as an antioxidant and a prooxidant. Notably, its prooxidant activity occurs specifically in cancer cells such as A549 and PC9, which typically exhibit high glycolytic levels [40]. As a powerful antoxidant, LA has been used in the treatment of chronic disease associated with high oxidative stress, including diabetic polyneuropathy and Alzheimer′s disease [59, 60]. In our previous work, we found that LA acts as a prooxidant at high doses (IC50 = 3–6 mM) to exert anticancer effects [40]. In the present study, low‐dose LA (200–400 μM) effectively inhibited inflammatory cytokine secretion. Therefore, low doses of LA may be suitable for preventing inflammation‐induced carcinogenesis. The dosage used in mice (91 mg/kg) in this study was converted from the clinical human dose (600 mg/day). Although LA is already used in clinical treatments, the bioavailability and safety profiles still require further evaluation.

This study has several limitations that should be considered when interpreting the findings. First, while the use of the RAW264.7 macrophage cell line provides a controlled model, it may not fully recapitulate the behavior of primary macrophages or the complexity of the in vivo tumor microenvironment. Future studies employing primary cells or more complex co‐culture systems would strengthen the conclusions. Second, although our in vivo data are promising, an ALI model was used to investigate the role of LA in chronic inflammation. This model does not fully capture the relationship between metabolic reprogramming and inflammation during the progressive development of inflammation‐induced carcinogenesis. In future study, an animal chronic inflammation model should be developed. Moreover, this study does not fully establish the therapeutic potential of LA for established inflammatory conditions. Further investigations exploring optimal therapeutic dosing windows and multiple administration schedules are warranted. Finally, while our data strongly suggest a causal pathway where LA inhibits inflammation by targeting HIF‐1α‐mediated glycolysis, the evidence is primarily based on pharmacological inhibition. To definitively establish causality, future research should employ genetic approaches, such as HIF‐1α knockdown or knockout animal models, to confirm that the anti‐inflammatory effects of LA are entirely dependent on this specific pathway.

In the inflammatory microenvironment, macrophages secrete inflammatory factors. If we can promptly and reversibly modulate the secretory state of macrophages—particularly by altering their metabolic patterns to shift the inflammatory profile—this approach could prevent both cytokine storms and carcinogenesis resulting from a persistently inflamed microenvironment. Such a strategy holds significant clinical promise.

## 5 Conclusions

The results of this study demonstrate that LA acts as an HIF1α inhibitor that suppresses macrophage polarization toward glycolysis, thereby preventing the release of inflammatory factors. By targeting the HIF1α/glycolysis axis, LA exhibited anti‐inflammatory effects in both LPS‐induced RAW264.7 cells and an ALI animal model. Overall, inhibitors of macrophage glycolysis show promising anti‐inflammatory properties. These findings provide a new foundation for the clinical prevention of cancer associated with chronic inflammation.

## Author Contributions

**Liduo Yue, Kai Wang:** conceived and designed the analysis. **Kai Wang, Liduo Yue:** collected the data. **Liduo Yue, Rongyuan Wang:** contributed data or analysis tools. **Kai Wang, Linbei Lu and Guoshu Li:** performed the analysis. **Liduo Yue, Lihong Fan:** wrote the paper.

## Conflicts of Interest

The authors declare no conflicts of interest.

## Data Availability

The datasets used and/or analyzed during the current study are available from the corresponding author on reasonable request.
